# The Psychometric Properties of a Short UPPS-P Impulsive Behavior Scale Among Psychiatric Patients Evaluated in an Emergency Setting

**DOI:** 10.3389/fpsyt.2019.00139

**Published:** 2019-03-25

**Authors:** Jules Roger Dugré, Charles-Édouard Giguére, Olivier Percie du Sert, Stephane Potvin, Alexandre Dumais, Consortium Signature

**Affiliations:** ^1^Department of Psychiatry, Institut Universitaire en Santé Mentale de Montréal, Montreal, QC, Canada; ^2^Département de Psychiatrie et d'addictologie, Faculté de Médecine, Université de Montréal, Montreal, QC, Canada; ^3^Department of Psychiatry, Institut National de Psychiatrie légale Philippe-Pinel, Montreal, QC, Canada

**Keywords:** impulsivity, short version, reliability, validity, psychiatric emergency

## Abstract

**Objective:** Impulsivity is a multidimensional construct that has an important role for the understanding of diverse psychopathologies and problematic behaviors. The UPPS-P impulsive behavior scale, measuring five distinct facets of impulsivity, has been subject to several studies. No study has investigated the clinical utility of this questionnaire amongst an unstable psychiatric population. The aim of the current study is to examine the psychometric properties of the short version of this scale in a psychiatric emergency unit.

**Method:** The S-UPPS-P was administered to 1,097 psychiatric patients in an emergency setting, where a subgroup of 148 participants completed a follow-up. The internal consistency, the construct validity, the test-retest reliability, and correlations with a substance misuse measure were examined.

**Results:** Confirmatory factor analyses supported a five-factor solution. Results indicated good psychometric properties across psychiatric diagnoses and gender. The S-UPPS-P was partially invariant across sexes. The authors have found differences on the loading of one item and on the thresholds of two items from lack of premeditation and positive urgency subscales.

**Conclusion:** This validation study showed that the UPPS-P conserved good psychometric properties in an unstable psychiatric sample, indicating that the instrument can be utilized in such settings.

## Introduction

The nature of impulsivity, now known to be heterogeneous (1), is generally characterized by deficits in delaying gratification, impulse and urge control, decision making, and maladaptive behaviors (2). Its cross-cutting nosology component, in line with the Research Domain Criteria framework (e.g., RDoC Cognitive Systems) (3, 4), plays a major role in the understanding of diverse psychopathologies and problematic behaviors. In fact, it is not surprising that impulsivity is one of the most common diagnostic criteria in the fifth edition of the *Diagnostic and Statistical Manual for Mental Disorders* (5) (e.g., Attention Deficit Hyperactivity Disorder and Conduct Disorder, Borderline Personality Disorder, Antisocial Personality Disorder, Substance Use Disorders, Bipolar Disorder).

However, it is interesting to note that impulsivity has been subject to terminological and conceptual confusion. To clarify the jingle-jangle fallacy^1^ of impulsivity (6), exploratory and confirmatory factor analyses have been performed using the Urgency-Premeditation-Perseverance-Sensation Seeking-Positive Urgency (UPPS-P) impulsive behavior scale which revealed five specific facets of impulsivity (7). These dimensions are *sensation seeking* (tendency to seek out novel and thrilling experiences), *lack of premedita*tion (tendency to not take into account the consequences of actions), *lack of perseverance* (tendency to have difficulty staying focused on a task that can be long, boring or difficult), *negative urgency* (the tendency to act rashly while in an intense negative mood), and *positive urgency* (the tendency to act rashly while in an intense positive mood).

Several studies have observed associations between the different dimensions of UPPS-P and distinct psychopathologies and problematic behaviors (e.g., substance abuse/dependence, aggressive and suicidal behaviors). In fact, *Lack of Premeditation* has been shown to be associated with substance misuse (8, 9), antisocial (ASPD) and borderline personality disorder (BPD) features (8, 10, 11), and violent behaviors (12, 13). *Lack of Perseverance* has been related to problematic substance use (8, 14), BPD characteristics (8, 10), and aggression (15). In regard to *Sensation Seeking*, studies have demonstrated a link with drug and alcohol use, gambling, ASPD traits, and delinquency (16, 17). *Positive Urgency* has been associated with illegal drug use and risky sexual behavior (18) as well as immediate gratification behaviors (19) and BPD traits (10). Lastly, *Negative Urgency* has been linked with problematic substance use (9), BPD traits (8, 10), depression and anxiety (8), suicidal behaviors (13), eating disorders (e.g., binging and purging) (8, 20), and aggression (8, 15).

Since the development of the UPPS-P model (16, 21, 22), recent studies found that the short 20-items version of the UPPS-P scale preserves its good psychometric properties (10, 23–26). More specifically, they showed, using various psychopathological scales, that the short version of the UPPS-P (S-UPPS-P) has acceptable to good internal consistency (Cronbach's alpha ranging from 0.61 to 0.88) (23–26); good to very good test-retest reliability (Correlation coefficients of ~0.87) (23), as well as similar factorial structure to the UPPS-P (23–26) and good external validity (23, 25, 26).

The transdiagnostic characteristic of impulsivity is highly relevant for clinicians regarding the understanding and the treatment of different forms of psychopathologies and problematic behaviors. In this sense, impulsivity constitutes a key target for clinical interventions (27). The UPPS-P model has attempted to clarify issues regarding the heterogeneity of impulsivity by presenting a strong and stable factorial structure. Considering the fact that the five dimensions of the UPPS-P are all specifically associated with distinct psychopathologies and problematic behaviors, the short version of the UPPS-P scale could be a suitable solution to overcome difficulties concerning the identification and the management of the broad spectrum of impulsivity-related problems in psychiatric patients. Along these lines, to our knowledge, no prior study has examined the psychometric properties of the UPPS-P scale amongst unstable psychiatric populations such as those in emergency department (ED). The objective of this current study is to examine the psychometric properties of the French version of the S-UPPS-P (23) in a large sample of adult psychiatric patients evaluated in a psychiatric ED. More precisely, we aimed to investigate the internal consistency, the construct validity, the test-retest reliability of the S-UPPS-P, as well as correlations between its subscales and a substance misuse measure, in this particular setting across psychiatric diagnoses and gender.

## Methodology

### Sample Description

The sample was taken from the Signature Bank of the *Institut Universitaire en Santé Mentale de Montréal* (IUSMM). Research nurses approached 1,862 eligible participants from the psychiatric emergency of the IUSMM. Of this number, 1,218 agreed to participate in the study. In the current validation study, 1,097 patients have accepted to participate. This is referred to as Time 1 (T1). French version of the S-UPPS-P used in the current study is taken from Bilieux et al. (23). These authors used forward-, consensus, and back-translation steps to assess the quality of the translation. Only 25 participants answered the questionnaire in the original English version. A subsample of 148 participants answered the questionnaire a second time within a 30-day interval right before leaving the hospital. This is referred to as T2. For details on the characteristics of patients at T1 and T2, please refer to Supplementary Tables 1, 2.

Our sample was mainly characterized by individuals with substance use disorders (SUDs) (*N* = 83), psychotic disorders (*N* = 429), mood disorders (*N* = 350), anxiety disorders (*N* = 104), personality disorders (PDs) (*N* = 115), and other psychiatric disorders (*N* = 16). Over half were men (*N* = 655 [59.7%]) with a mean (SD) age of 40.4 (14.1) years. All participants signed a detailed consent form, and the study was approved by the local ethics committee in accordance with the *Declaration of Helsinki*.

### Instruments

#### Psychiatric Diagnoses

Research nurses collected patient's psychiatric diagnoses from medical records. Psychiatric diagnoses were established by psychiatrists on the ward, and were coded according to the World Health Organization International Classification of Disease (ICD-10) (28). In this article, we used 6 of the categories of mental disorder (F00–F99): (1) Substance related disorders (F10-F19), (2) Schizophrenia and psychotic disorders (F20–F29), (3) Mood disorders (F30–F39), (4) Anxious disorders (F40–F49), (5) Personality Disorders (F60–F69), and (6) Others (F00–F09, F50–59, and F70–F99). As shown in Supplementary Table 1, more than 70% of patients were treated primarily for psychotic and mood disorders.

#### Impulsive Behavior Scale

The *Impulsive behavior scale* (S-UPPS-P) is an instrument composed of 20 items rated on a four-point Likert scale (23): (1) *disagree strongly*, (2) *disagree some*, (3) *agree some*, and (4) *agree strongly*. Five scales were computed by adding the 4 items corresponding to each scale: (1) negative urgency (NU), (2) positive urgency (PU), (3) sensation seeking (SS), (4) lack of perseveration (PE), and (5) lack of premeditation (PR). Each scale ranges from 4 to 16. The description of each scale by main psychiatric diagnosis and sex is indicated in Supplementary Table 3. The S-UPPS-P questionnaire is shown in Appendix 1.

#### Drug Abuse Screening Test (DAST-10)

The Drug abuse screening test is a 10-item check list. The items are dichotomous: (0) no, (1) yes. The total scale is the sum of the 10 items resulting in a scale of 0–10.

### Statistical Analyses

Analyses were all performed in R v3.3.0 (29). We used the *psych* package (30) for reliability analyses and the *lavaan* package (31) for Structural Equation modeling (SEM).

#### Internal Consistency

To evaluate the internal consistency of the S-UPPS-P, Cronbach alphas (32) were estimated for each of the five subscales: negative urgency (NU), positive urgency (PU), sensation seeking (SS), lack of perseveration (PE), lack of premeditation (PR). We tested first for all participants and then by sex and main psychiatric diagnosis.

#### Construct Validity

Following the work made by Billieux et al. (23), confirmatory factor analyses using a probit link were performed to confirm the structure of the S-UPPS-P. Four factorial models were compared: (1) a single-factor model (Model 1) in which all 20 items loaded on a unique “impulsivity factor,” (2) a five factor model (Model 2) representing the five subscales of the S-UPPS-P (see Supplementary Figure 1), (3) a three factor model (Model 3) represented by three latent variables: urgency, sensation seeking and lack of conscientiousness and this three factor model, adding hierarchical structure: urgency (positive and negative urgency as lower order factors), sensation seeking and lack of conscientiousness (lack of premeditation and perseverance as lower order factors). Membership in one of the 4 Likert categories was assigned using three thresholds and a latent variable for each items of the UPPS-P. Model were adjusted using lavaan package (31) in R (29). Group analyses by gender were examined to test for measurement invariance across sexes with ordered categorical variable (33).

#### Test-Retest Reliability

As described previously in the sample description, a subsample of 148 participants answered the UPPS-P questionnaire a second time just before their release from the hospital. Only the participants who answered the questionnaire within 30 days of their admissions were selected. The mean (± SD) time between emergency admission (T1) and hospital release (T2) was 14.1 ± 6.7 days. Correlations and intra-class correlations (ICC) were estimated to assess the stability of the impulsive behavior questionnaire.

#### Correlations With a Substance Misuse Measure (DAST-10)

An association has been regularly observed between SUDs and impulsivity, specifically sensation seeking (34). By using a confirmatory factor analysis, we tested for relationships between the impulsivity measures and substance use (as measured with the DAST-10), since both constructs are theoretically and empirically related.

#### Comparisons of Mean Differences of S-UPPS-P Subscales Between Diagnostic Categories

To better understand differences in S-UPPS-P subscales between diagnostic categories, we performed analysis of variance (ANOVA) *post hoc* pairwise comparisons tests, corrected with Tukey test for multiple comparisons.

## Results

### Internal Consistency

Cronbach's alpha for all the participants varied between 0.70 and 0.81 indicating an acceptable to a good reliability of the S-UPPS-P questionnaire. As illustrated in Table 1, each dropped item did not improve the Cronbach's alpha.

**Table 1:** Cronbach alpha for the full sample with 95% confidence interval.

**Negative Urgency**	**0.79 (0.77, 0.81)**	**Lack of Perseveration**	**0.81 (0.80, 0.83)**
With q4 dropped	0.76	With q5 dropped	0.80
With q7 dropped	0.73	With q8 dropped	0.73
With q12 dropped	0.73	With q11 dropped	0.75
With q17 dropped	0.75	With q16 dropped	0.78
Positive Urgency	0.70 (0.68, 0.73)	Lack of Premeditation	0.79 (0.76, 0.81)
With q2 dropped	0.64	With q1 dropped	0.75
With q10 dropped	0.69	With q6 dropped	0.73
With q15 dropped	0.60	With q13 dropped	0.72
With q20 dropped	0.62	With q19 dropped	0.74
Sensation Seeking	0.77 (0.75, 0.79)		
With q3 dropped	0.74		
With q9 dropped	0.69		
With q14 dropped	0.73		
With q18 dropped	0.69		

**Figure 1:**
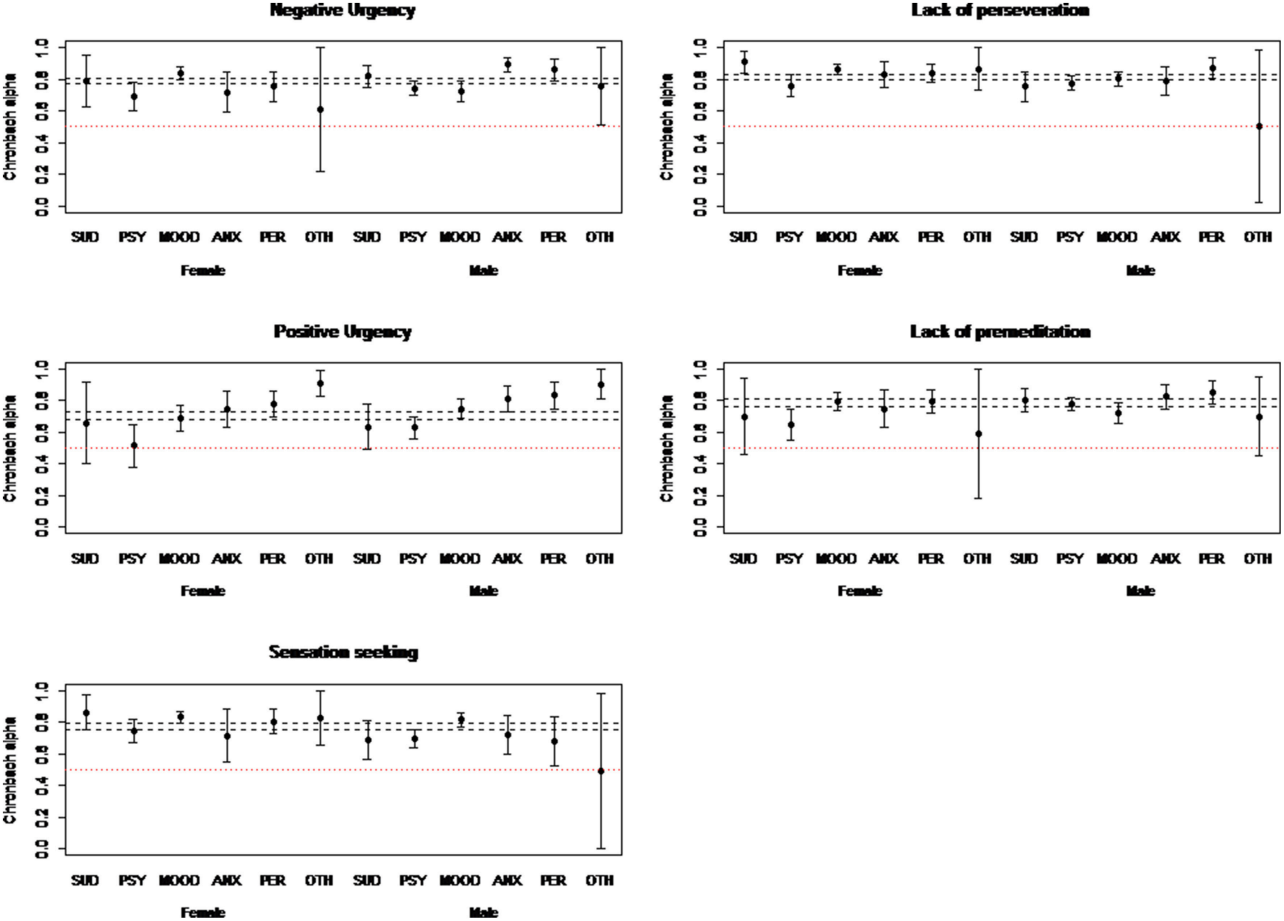
Cronbach alpha's intervals of S-UPPS-P subscales per diagnosis and sex (*n* = 1097).

Table 2 shows the results on the reliability of the UPPS-P by sex and main psychiatric diagnosis. In most subgroups, the 95% confidence intervals intersected with each other or are above the total alpha. Only the subgroup composed of women with psychotic disorders had a lower Cronbach's alpha. Figure 1 displays graphically the different confidence intervals. Some confidence intervals were very large due to a smaller prevalence of certain psychiatric disorders, such as SUDs and other psychiatric illnesses (F00–F09, F50–59, and F70–F99 in ICD-10). These results showed that the reliability of the UPPS is constant across psychiatric diagnoses and sexes.

**Table 2:** S-UPPS-P reliability (Cronbach α) by sex and main diagnostic.

**Main diagnostic**	**Female**	**Male**
**NEGATIVE URGENCY**		
Substance use	0.79 (0.63, 0.95)	0.82 (0.75, 0.89)
Psychotic disorder	0.69 (0.60, 0.78)	0.74 (0.70, 0.79)
Mood disorder	0.84 (0.80, 0.88)	0.73 (0.66, 0.79)
Anxious disorder	0.72 (0.59, 0.85)	0.90 (0.85, 0.94)
Personality disorder	0.76 (0.66, 0.85)	0.86 (0.79, 0.93)
Other	0.63 (0.22, 1.00)	0.77 (0.51, 1.00)
**POSITIVE URGENCY**		
Substance use	0.66 (0.40, 0.92)	0.63 (0.49, 0.78)
Psychotic disorder	0.51 (0.38, 0.65)	0.63 (0.56, 0.70)
Mood disorder	0.69 (0.61, 0.77)	0.75 (0.69, 0.81)
Anxious disorder	0.75 (0.63, 0.86)	0.81 (0.73, 0.89)
Personality disorder	0.78 (0.70, 0.86)	0.83 (0.75, 0.92)
Other	0.91 (0.83, 0.99)	0.91 (0.81, 1.00)
**SENSATION SEEKING**		
Substance use	0.86 (0.75, 0.97)	0.69 (0.56, 0.81)
Psychotic disorder	0.74 (0.67, 0.82)	0.70 (0.64, 0.75)
Mood disorder	0.83 (0.79, 0.87)	0.81 (0.77, 0.86)
Anxious disorder	0.69 (0.55, 0.88)	0.72 (0.60, 0.84)
Personality disorder	0.81 (0.73, 0.88)	0.67 (0.52, 0.83)
Other	0.84 (0.65, 1.00)	0.37 (0.00, 0.98)
**LACK OF PERSEVERATION**		
Substance use	0.91 (0.84, 0.98)	0.76 (0.66, 0.85)
Psychotic disorder	0.76 (0.69, 0.83)	0.77 (0.73, 0.82)
Mood disorder	0.87 (0.83, 0.90)	0.81 (0.76, 0.85)
Anxious disorder	0.83 (0.75, 0.91)	0.79 (0.70, 0.88)
Personality disorder	0.84 (0.78, 0.90)	0.87 (0.81, 0.94)
Other	0.87 (0.73, 1.00)	0.51 (0.02, 0.99)
**LACK OF PREMEDITATION**		
Substance use	0.70 (0.46, 0.94)	0.81 (0.73, 0.88)
Psychotic disorder	0.65 (0.55, 0.75)	0.78 (0.74, 0.82)
Mood disorder	0.79 (0.74, 0.85)	0.73 (0.66, 0.79)
Anxious disorder	0.75 (0.63, 0.87)	0.82 (0.75, 0.90)
Personality disorder	0.80 (0.72, 0.87)	0.86 (0.78, 0.93)
Other	0.63 (0.18, 1.00)	0.70 (0.45, 0.95)

### Construct Validity

Table 3 demonstrates the results of the 4 model comparisons. The best fitting model using CFI, TLI, RMSEA and SRMR was obtained with the five-factor model (Model 2). This model had a good fit (35), CFI = 0.98 > 0.90 and the upper part of the 90% confidence interval of the RMSEA was lower than 0.08, RMSEA 90% C.I. = (0.065, 0.073). The three-factor hierarchical model (Model 4) also showed good fit of the data (RMSEA = 0.082 (CI: 0.078–0.086), CFI = 0.97, TLI = 0.96 SRMR = 0.078). As shown in Table 3, the two other models provided worse model fits.

**Table 3:** Comparison of the 4 factorial models.

**Model**	**CFI**	**TLI**	**RMSEA (90% C.I.)**	**SRMR**
1	0.765	0.738	0.209 (0.205, 0.213)	0.170
2	0.976	0.971	0.069 (0.065, 0.073)	0.066
3	0.948	0.941	0.100 (0.096, 0.103)	0.089
4	0.965	0.959	0.082 (0.078, 0.086)	0.078

**Figure 2:**
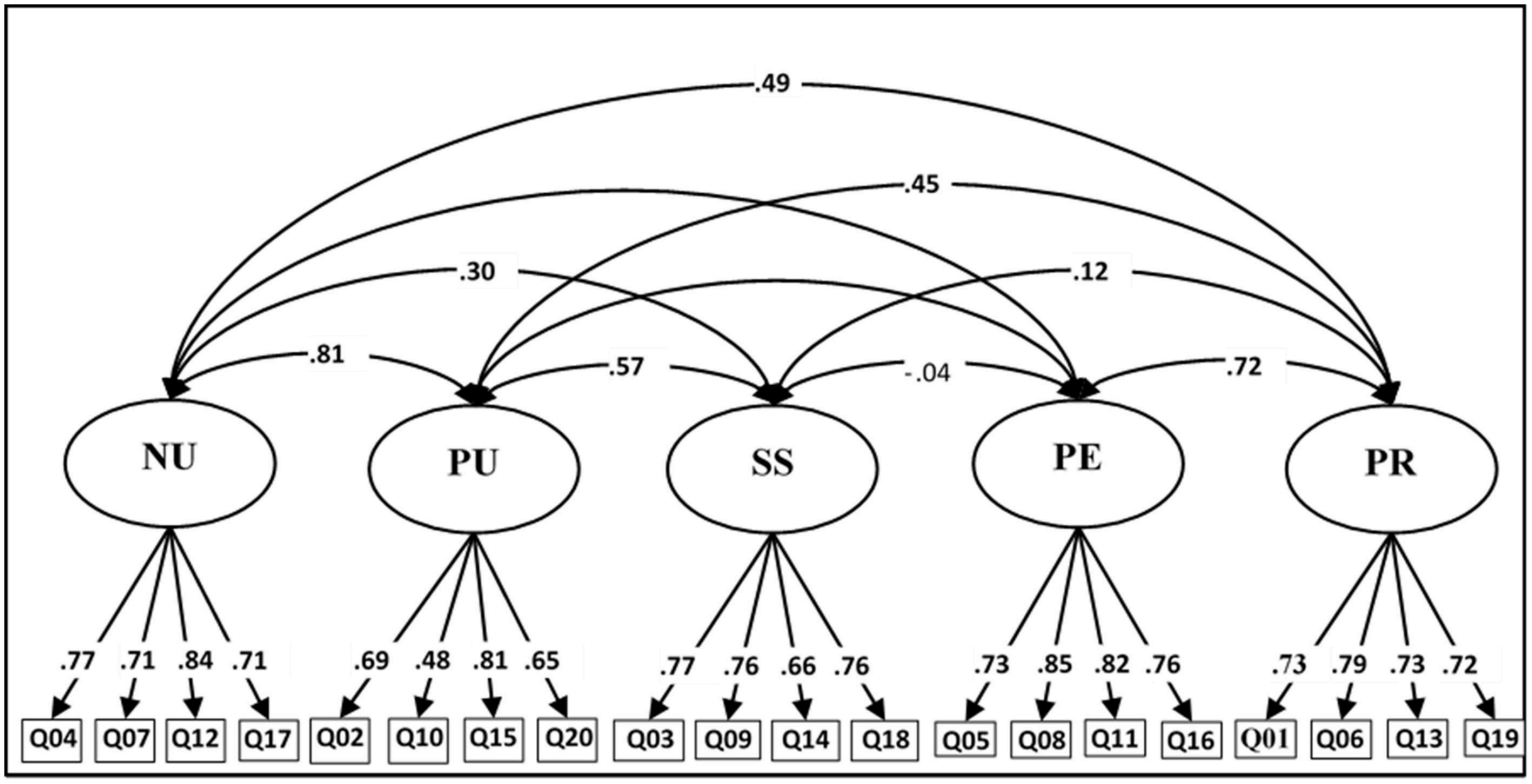
Five-factor model structure of the S-UPPS-P.

The 5-factor model, as shown in Figure 2, is formed by negative urgency (NU), positive urgency (PU), sensation seeking (SS), lack of perseverance (PE) as well as lack of premeditation (PR). We also performed a multi-group analysis by sex to test for measurement invariance. Measurement invariance was tested for sex using the procedure by Millsap and Yun-Tein (33) for ordered categorical variable. First comparison between the configural model and the equal loadings model using scaled chi-squared differences (36) gave a statistically significant differences Δ$\chi_{\left(15\right)}^{2}$ = 27.2, *p* = 0.0275 but the difference in CFI and TLI was very small (~0.001). By freeing loadings of item 6 (PR: “My thinking is usually careful and purposeful”), and item 10 (PU: “When overjoyed, I feel like I can't stop myself from going overboard”) the difference with the configural models were no longer statistically significant Δ$\chi_{\left(18\right)}^{2}$ = 18.1, *p* = 0.1533. When we compared the partially freed loadings model to the fixed thresholds model the difference was statistically significant Δ$\chi_{\left(35\right)}^{2}$ = 67.3, *p* < 0.001. We stopped invariance testing at this step because of the large chi-square differences. The difference was mostly driven by the sex difference on the second and third threshold of item 10 which was respectively 0.333 and 0.346. This indicates that men over reported item 10 even when conditioned on the positive urgency factor.

### Test-Retest Reliability

Some participants were followed through their clinical pathways. We analyzed responses from participants who answered within 30 days of their original assessment just before they were released from the hospital. Results are presented in Table 4. The total score had a good test-retest reliability according to the correlation and intra-class correlation coefficients which were both around 0.60 and above. The scores ranged from 0.4 to 1.0 unit lower at Time 2.

**Table 4:** Test-retest reliability (*N* = 148) for the impulsive behavior score within 30 days.

**Measure**	**(T1) mean ± sd**	**(T2) mean ± sd**	***r* (T1, T2)**	**ICC (T1, T2)**
Negative urgency	10.3 ± (3.4)	9.1 ± (3.0)	0.61^***^	0.56^***^
Positive urgency	11.0 ± (3.0)	10.1 ± (2.7)	0.60^***^	0.57^***^
Sensation seeking	9.7 ± (3.3)	9.2 ± (3.2)	0.64^***^	0.63^***^
Lack of perseveration	7.4 ± (2.8)	7.2 ± (2.7)	0.66^***^	0.66^***^
Lack of premeditation	7.6 ± (2.7)	7.2 ± (2.4)	0.65^***^	0.64^***^

* p < 0.05

** p < 0.01

*** *p < 0.001*.

### Correlations With a Substance Misuse Measure (DAST-10)

**Figure 3:**
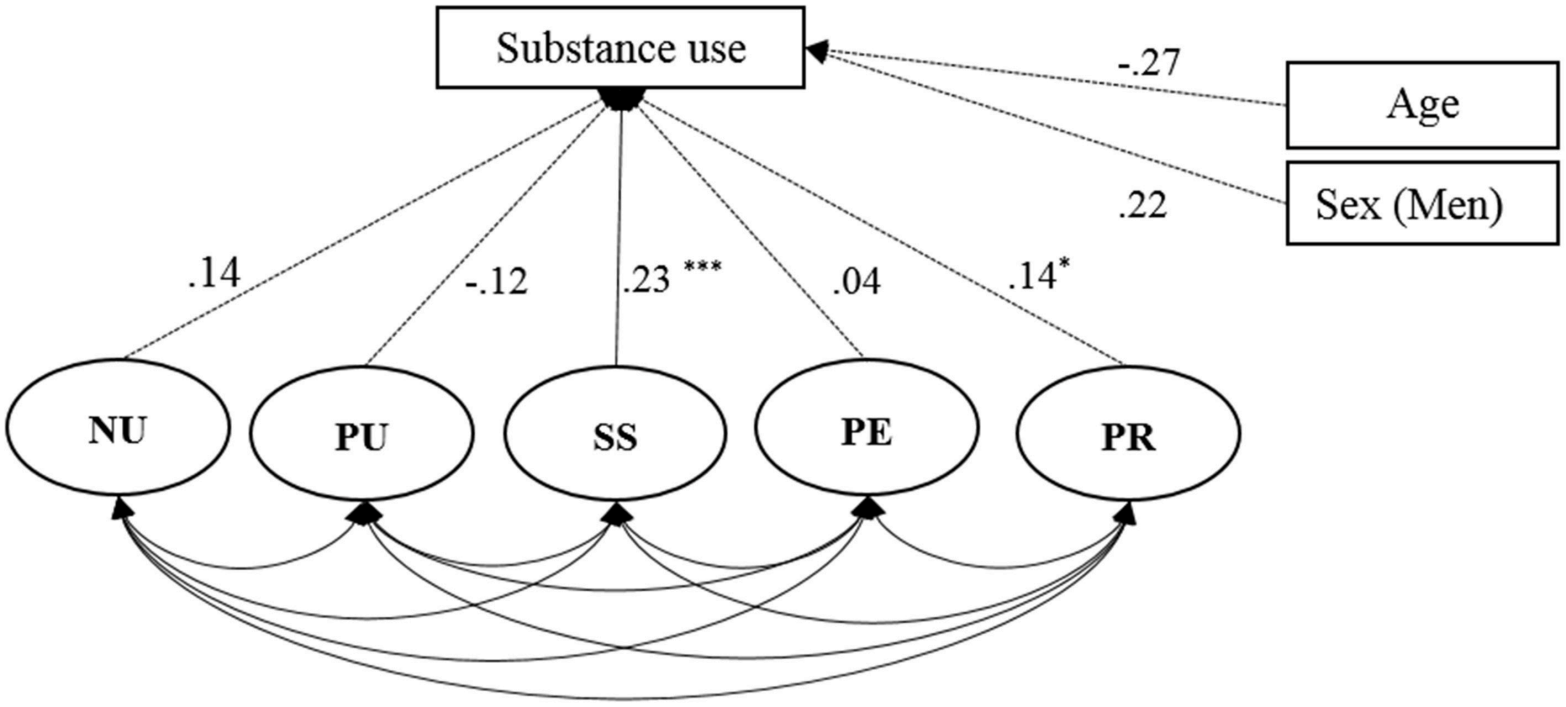
Results of the confirmatory factor analysis (CFA) to test the link between substance use (DAST-10) and the impulsive behavior questionnaire (S-UPPS-P) dimensions: negative urgency (NU), positive urgency (PU), lack of perseveration (PE), lack of premeditation (PR); Age and Sex are added as adjustment variables; * <0.05; ** <0.01; *** <0.001.

We tested correlations between the five subscales of the S-UPPS-P and a substance use measure (DAST-10). We expected that patient's impulsivity score, specifically the sensation seeking subscale score, would be associated with the DAST total score. This was tested using a CFA with the factor structure described earlier. Results of the CFA of the 5 dimensions of the S-UPPS-P regressed on the DAST-10 showed that sensation seeking correlated with substance use (r = 0.37, *p* < 0.001). A trend was observed concerning a positive association between Lack of premeditation and substance use (*r* = 0.13, *p* = 0.053). These results remained statistically significant after the inclusion of age and sex as adjustment variables (see Figure 3).

### Comparisons of Mean Differences of S-UPPS-P Subscales Between Diagnostic Categories

Results suggest significant differences between diagnostic categories across every S-UPPS-P subscale, as shown in Table 5. More specifically, pairwise *post hoc* tests showed that participants with a PD scored significantly higher in Negative Urgency, Positive Urgency, Sensation Seeking, Lack of Premeditation, and Lack of Perseverance subscales than almost every other diagnostic category (see Supplementary Table 4). In comparison to patients with a SUD, those with a PD scored significantly higher only on Lack of Premeditation (*p* = 0.002) and Lack of Perseverance (*p* = 0.019).

**Table 5:** Comparisons of mean differences of S-UPPS-P subscales between diagnostic categories (*n* = 1,097).

**S-UPPS-P subscales**	**Substance use disorders (*n* = 83)**	**Psychotic disorders (*n* = 429)**	**Mood disorders (*n* = 350)**	**Anxiety disorders (*n* = 104)**	**Personality disorders (*n* = 115)**	**Other disorders (*n* = 16)**	***F-Statistics***
							
NU	12.17 (3.09)	9.85 (3.21)	10.38 (3.30)	11.52 (3.31)	13.02 (2.86)	11.69 (3.40)	23.82^***^
PU	12.04 (2.62)	10.43 (2.84)	10.67 (3.04)	11.03 (3.19)	12.56 (3.00)	12.12 (3.72)	12.82^***^
SS	11.14 (2.92)	9.53 (3.24)	9.69 (3.53)	8.77 (2.91)	10.55 (3.18)	9.56 (3.10)	6.66^***^
PR	8.25 (2.86)	7.26 (2.69)	7.40 (2.67)	8.09 (2.79)	9.74 (3.01)	7.69 (2.52)	16.86^***^
PE	7.70 (3.01)	7.70 (3.01)	7.49 (3.05)	7.71 (2.88)	9.03 (3.21)	7.12 (2.45)	16.86^***^

Means and standard deviations are reported for each S-UPPS-P subscale by diagnostic categories. NU, Negative Urgency; PU, Positive Urgency; SS, Sensation Seeking; PR, Lack of Premeditation; PE, Lack of Perseverance; *p < 0.05; **p < 0.01;

*** *p < 0.001*.

## Discussion

The present study examined the psychometric properties of the S-UPPS-P questionnaire (23) which was administered in a psychiatric ED. Generally, results demonstrated that the S-UPPS-P has good psychometric properties across psychiatric diagnoses and sexes in this specific setting. Moreover, Cronbach' alphas supported acceptable to good internal consistency within dimensions: negative urgency (0.79), positive urgency (0.70), lack of perseveration (0.81), lack of premeditation (0.79), and sensation seeking (0.77). The internal consistency reliability was found to be constant across psychiatric diagnoses and sexes but was lower in the subgroup composed of women with psychotic disorders. The reasons for this latter observation are elusive. Furthermore, CFA confirmed similar structures consistent with previous studies (five-factor model: 25, 26 and three-factor hierarchical model: 23). Correlations and intra-class correlation coefficients indicated that the S-UPPS-P had a good test-retest reliability. While below the usual cut-off (>0.70), results were good considering the psychological instability of patients admitted in a psychiatric ED (T1) in comparison to their release (T2). Finally, by testing correlations with a substance misuse measure, we observed a strong association between substance use and sensation seeking. These latter findings are similar to those of previous studies on impulsivity in SUD populations (34), and further justify the use of factors rather the use of a broad UPPS total score. Additionally, our results suggest that the distinction between impulsive behavior factors seems to be determinant in the comprehension of specific psychopathology and inadequate behaviors such as drug misuse.

A significant finding in our study is that we were able to replicate the validity of the theory-driven factor structure of the UPPS-P model across both psychiatric diagnoses and gender within a psychiatric ED. Indeed, both structures has been identified in general population samples using French (23), English (25), Spanish (24), and Italian (26) versions of the S-UPPS-P. The five distinct, yet interrelated factors (positive urgency, negative urgency, lack of premeditation, lack of perseverance, and sensation seeking) have already shown valuable implications for psychiatric research since each dimension was strongly associated with specific psychopathologies. In fact, our study has shown that participants with a PD had significantly higher scores than those with a psychotic, a mood, or an anxiety disorder on almost every subscales of the S-UPPS-P, thus reflecting that impulsivity and its subcomponents are core features of personality disorders (8, 10, 16). However, patients with PD did not significantly differ from patients with a SUD on the sensation seeking or the urgency's subscales. In fact, earlier studies suggested that impulsivity is a shared risk factor for developing a PD and/or a SUD (37, 38). While PDs and SUDs may share subcomponents of impulsivity, the non-statistically significant results could be explained by the fact that both disorders are highly heterogeneous and intercorrelated (39, 40). Thus, different subtypes of individuals with a PD and/or a SUD may be associated with distinct subcomponents of impulsivity (41, 42). Nonetheless, our results suggest that the short version of the UPPS-P scale preserved its good psychometric properties in a sample of unstable psychiatric patients. Therefore, research on psychiatric unstable patients could use the S-UPPS-P to better understand psychopathologies and their comorbidities.

While the S-UPPS-P displayed overall psychometric properties that were satisfactory, we found that the construct validity of the instrument was only weakly invariant across sexes. Although this result needs to be interpreted cautiously, it may have implications for future studies on the psychometric properties of the UPPS as well as on gender differences in impulsivity. Recently, a meta-analysis found that there were gender differences in impulsivity (43). Nevertheless, it has been subsequently argued by Cyders (44) that conclusions concerning gender differences in impulsivity might be premature, because the measurement invariance of impulsivity scales across sexes is largely unknown. This author investigated this issue regarding the UPPS-P in a sample of undergraduate and found a measurement invariance across sexes but found a higher reported level of sensation seeking in men when compared to woman (44). Our results suggest that the S-UPPS-P is partially invariant across sexes when administered to psychiatric patients in an ED. More specifically, we found that item 10 of the positive urgency subscale was overrated by men. This suggest that, in this particular setting, there could be a bias in positive urgency response between sexes. Therefore, future studies on sex differences regarding impulsivity should pay special attention to these subtle methodological issues.

Limitations in the current study need to be acknowledged. First, psychiatrist on the ward did not establish the diagnosis of psychiatric disorders with a validated instrument such as the Structure Clinical Interview for DSM-V. Also, we did not include another impulsivity scale (e.g., Barratt Impulsiveness Scale), nor measures related to the five subscales of the UPPS-P model. Therefore, future studies should seek to test the convergent validity of the UPPS-P. Finally, due to small sample sizes across diagnostic categories, we did not examine measurement invariance across diagnostic categories. However, this should be considered in future studies in order to assess the stability of the psychometric properties of the S-UPPS-P across psychiatric disorders.

On the other hand, the strengths of the current study include the administration of the S-UPPS-P to a large sample of patients in a specific clinical environment (e.g., psychiatric ED), the examination of several psychometric properties (e.g., internal consistency, construct validity, test-retest reliability, and correlations with a substance misuse measure), and the verification of the measurement invariance of the instrument across and sexes.

## Conclusion

In conclusion, the current study showed that the S-UPPS-P is a valid questionnaire for psychiatric research in general, and for research on unstable psychiatric population (e.g., psychiatric ED). However, researchers should be aware that the S-UPPS-P might not be the scale with the most optimal psychometric properties for evaluating gender differences in sensation seeking.

## Author Contributions

AD and SP designed the study and wrote the protocol. JD and OP managed the literature searches. C-ÉG undertook the statistical analysis. JD wrote the first draft of the manuscript. All authors contributed to and have approved the final manuscript.

### Conflict of Interest Statement

The authors declare that the research was conducted in the absence of any commercial or financial relationships that could be construed as a potential conflict of interest.

## Footnotes

^1^The “jingle” fallacy refers to an assumption that two different constructs are the same because they have the same label; the “jangle” fallacy refers to an assumption that two identical constructs are different because they are labeled differently
