# Supplementary material for: The Psychometric Properties of a Short UPPS-P Impulsive Behavior Scale Among Psychiatric Patients Evaluated in an Emergency Setting
Source: Front Psychiatry. 2019 Mar 25;10:139. doi: 10.3389/fpsyt.2019.00139 (PMC6442540; doi:10.3389/fpsyt.2019.00139)
Supplement: Supplementary file 1 [file Data_Sheet_1.docx]

# **Supplementary Tables & Figures**

**Supplementary Table 1:** Characteristics of patients (n=1097) from Signature Bank

|  |  | Female | Male |
| --- | --- | --- | --- |
| Age |  | 442 (40.3%) | 655 (59.7%) |
| 18-19 years | 48 (4.4%) | 19 (39.6%) | 29 (60.4%) |
| 20-29 years | 250 (22.8%) | 92 (36.8%) | 158 (63.2%) |
| 30-39 years | 272 (24.8%) | 100 (36.8%) | 172 (63.2%) |
| 40-49 years | 197 (18.0%) | 76 (38.6%) | 121 (61.4%) |
| 50-59 years | 214 (19.5%) | 95 (44.4%) | 119 (55.6%) |
| 60-81 years | 116 (10.6%) | 60 (51.7%) | 56 (48.3%) |
|  |  | Female | Male |
| Main diagnostic |  | 442 (40.3%) | 655 (59.7%) |
| Substance use | 83 (7.6%) | 17 (20.5%) | 66 (79.5%) |
| Psychotic disorder | 429 (39.1%) | 130 (30.3%) | 299 (69.7%) |
| Mood disorder | 350 (31.9%) | 169 (48.3%) | 181 (51.7%) |
| Anxious disorder | 104 (9.5%) | 45 (43.3%) | 59 (56.7%) |
| Personality disorder | 115 (10.5%) | 74 (64.3%) | 41 (35.7%) |
| Other disorder | 16 (1.5%) | 7 (43.8%) | 9 (56.2%) |
|  |  |  |  |

**Supplementary Table 2:** Characteristics of patients (n=148) from Signature Bank who answered UPPS-P at T2

|  |  | Female | Male |
| --- | --- | --- | --- |
| Age |  | 63 (42.6%) | 85 (57.4%) |
| 18-19 years | 9 (6.1%) | 1 (11.1%) | 8 (88.9%) |
| 20-29 years | 34 (23.0%) | 11 (32.4%) | 23 (67.6%) |
| 30-39 years | 26 (17.6%) | 7 (26.9%) | 19 (73.1%) |
| 40-49 years | 26 (17.6%) | 13 (50.0%) | 13 (50.0%) |
| 50-59 years | 36 (24.3%) | 20 (55.6%) | 16 (44.4%) |
| 60-81 years | 17 (11.5%) | 11 (64.7%) | 6 (35.3%) |
|  |  | Female | Male |
| Main diagnostic |  | 63 (42.6%) | 85 (57.4%) |
| Substance use | 9 (6.1%) | 1 (14.3%) | 6 (85.7%) |
| Psychotic disorder | 34 (23.0%) | 15 (30.6%) | 34 (69.4%) |
| Mood disorder | 26 (17.6%) | 38 (48.7%) | 40 (51.3%) |
| Anxious disorder | 26 (17.6%) | 6 (66.7%) | 3 (33.3%) |
| Personality disorder | 36 (24.3%) | 3 (60.0%) | 2 (40.0%) |
| Other disorder | 17 (11.5%) | 0 (0.0%) | 0 (0.0%) |

**Supplementary Table 3:** Description (mean ± SD) of the impulsive behavior scale by main psychiatric diagnosis and sex.

| Sex | Main psychiatric diagnosis | NU | PU | SS |
| --- | --- | --- | --- | --- |
| Female | Substance use | 12.5 ± (2.9) | 12.1 ± (2.8) | 10.5 ± (3.1) |
|  | Psychotic disorder | 9.6 ± (3.2) | 10.4 ± (2.7) | 8.7 ± (3.3) |
|  | Mood disorder | 10.8 ± (3.5) | 10.9 ± (3.0) | 9.2 ± (3.6) |
|  | Anxious disorder | 11.4 ± (3.2) | 10.3 ± (3.1) | 7.6 ± (2.4) |
|  | Personality disorder | 13.2 ± (2.6) | 13.0 ± (2.9) | 10.6 ± (3.4) |
|  | Other | 11.9 ± (3.1) | 11.7 ± (3.8) | 8.0 ± (2.8) |
| Male | Substance use | 12.1 ± (3.2) | 12.0 ± (2.6) | 11.3 ± (2.9) |
|  | Psychotic disorder | 10.0 ± (3.2) | 10.4 ± (2.9) | 9.9 ± (3.2) |
|  | Mood disorder | 10.0 ± (3.0) | 10.5 ± (3.1) | 10.1 ± (3.4) |
|  | Anxious disorder | 11.6 ± (3.4) | 11.6 ± (3.2) | 9.7 ± (2.9) |
|  | Personality disorder | 12.6 ± (3.3) | 11.8 ± (3.1) | 10.5 ± (2.8) |
|  | Other | 11.6 ± (3.8) | 12.4 ± (3.8) | 10.8 ± (2.9) |
|  |  |  |  |  |
| Sex | Main diagnostic | PE | PR |  |
| Female | Substance use | 8.4 ± (3.2) | 9.0 ± (2.6) |  |
|  | Psychotic disorder | 6.7 ± (2.6) | 7.0 ± (2.5) |  |
|  | Mood disorder | 7.7 ± (3.3) | 7.7 ± (2.9) |  |
|  | Anxious disorder | 8.0 ± (3.0) | 8.3 ± (2.6) |  |
|  | Personality disorder | 9.1 ± (3.2) | 10.1 ± (3.0) |  |
|  | Other | 6.9 ± (2.7) | 8.3 ± (2.0) |  |
| Male | Substance use | 7.5 ± (3.0) | 8.0 ± (2.9) |  |
|  | Psychotic disorder | 7.5 ± (3.0) | 7.4 ± (2.8) |  |
|  | Mood disorder | 7.3 ± (2.8) | 7.2 ± (2.4) |  |
|  | Anxious disorder | 7.5 ± (2.8) | 7.9 ± (2.9) |  |
|  | Personality disorder | 8.8 ± (3.2) | 9.2 ± (2.9) |  |
|  | Other | 7.3 ± (2.3) | 7.2 ± (2.9) |  |

NU: Negative urgency, PU: Positive urgency, SS: Sensation seeking, PE: Lack of perseveration, and PR: Lack of premeditation.

Q4

Q7

Q17

Q12

Q2

Q10

Q20

Q15

Q5

Q8

Q16

Q11

Q1

Q6

Q19

Q13

Q3

Q9

Q18

Q14

**Supplementary Figure 1** Confirmatory factor analysis model of the S-UPPS-P (Billieux et al. 2012)

| **Supplementary Table 4:** Post Hoc Comparisons of mean differences of S-UPPS-P subscales between diagnostic categories (n=1097) | | | | | | |
| --- | --- | --- | --- | --- | --- | --- |
| Diagnostic Category 1 | Diagnostic Category 2 | **NU** (p-value) | **PU** (p-value) | **SS** (p-value) | **PR** (p-value) | **PE** (p-value) |
|  |  |  |  |  |  |  |
|  |  |  |  |  |  |  |
| Substance Use Disorders | Psychotic Disorders | **<0.001** | **<0.001** | **<0.001** | **0.027** | N.S |
|  | Mood Disorders | **<0.001** | **0.002** | **0.003** | N.S | N.S |
|  | Anxiety Disorders | N.S | N.S | **<0.001** | N.S | N.S |
|  | Personality Disorders | N.S | N.S | N.S | **0.002** | **0.019** |
|  | Other Disorders | N.S | N.S | N.S | N.S | N.S |
| Psychotic Disorders | Mood Disorders | N.S | N.S | N.S | N.S | N.S |
|  | Anxiety Disorders | **<0.001** | N.S | N.S | N.S | N.S |
|  | Personality Disorders | **<0.001** | **<0.001** | **0.031** | **<0.001** | **<0.001** |
|  | Other Disorders | N.S | N.S | N.S | N.S | N.S |
| Mood Disorders | Anxiety Disorders | 0.015 | N.S | N.S | N.S | N.S |
|  | Personality Disorders | **<0.001** | **<0.001** | N.S | **<0.001** | **<0.001** |
|  | Other Disorders | N.S | N.S | N.S | N.S | N.S |
| Anxiety Disorders | Personality Disorders | **0.006** | **0.002** | **<0.001** | **<0.001** | **0.011** |
|  | Other Disorders | N.S | N.S | N.S | N.S | N.S |
| Personality Disorders | Other Disorders | N.S | N.S | N.S | **0.049** | N.S |
| *Note.* Results reported were corrected using Tukey test for multiple comparisons. NU = Negative Urgency; PU=Positive Urgency; SS=Sensation Seeking; PR=Lack of Premeditation; PE=Lack of Perseverance; N.S = Non-Significant | | | | | | |
|  |  |  |  |  |  |  |

**Appendix 1: Impulsive behavior scale (S-UPPS-P)**

1. I usually think carefully before doing anything [PR]
2. When I am really excited, I tend not to think on the consequences of my actions [PU]
3. I sometime like doing things that are a bit frightening [SS]
4. When I am upset I often act without thinking [NU]
5. I generally like to see things through to the end [PE]
6. My thinking is usually careful and purposeful [PR]
7. In the heat of an argument, I will often say things that I later regret [NU]
8. I finish what I start [PE]
9. I quite enjoy taking risks [SS]
10. When overjoyed, I feel like I can't stop myself from going overboard [PU]
11. Once I start a project, I almost always finish it [PE]
12. I often make matters worse because I act without thinking when I am upset [NU]
13. I usually make up my mind through careful reasoning [PR]
14. I generally seek new and exciting experiences and activities [SS]
15. I tend to act without thinking when I am really excited [PU]
16. I am a productive person who always gets the job done [PE]
17. When I feel rejected, i will often say things that I later regret [NU]
18. I welcome new and exciting experiences and sensations, even if they are a little frightening and unconventional [SS]
19. Before making up my mind, I consider all the advantages and disadvantages [PR]
20. When I am very happy, I feel like it is OK to give into cravings or overindulge [PU]
